# Activities and Perceived Risk of Transmission and Spread of SARS-CoV-2 among Specialists and Residents in a Third Level University Hospital in Spain

**DOI:** 10.3390/ijerph18062838

**Published:** 2021-03-10

**Authors:** Jesús María Aranaz-Andrés, Amaranta McGee-Laso, Juan Carlos Galán, Rafael Cantón, José Mira, on behalf of the Team of Work COVID-19

**Affiliations:** 1 Servicio de Medicina Preventiva y Salud Pública, Hosital Universitario Ramón y Cajal, 28034 Madrid, Spain; jesusmaria.aranaz@salud.madrid.org; 2 Instituto Ramón y Cajal de Investigación Sanitaria (IRYCIS), 28034 Madrid, Spain; 3 Facultad de Ciencias de la Salud, Universidad Internacional de la Rioja, 26006 Logroño, Spain; 4 CIBER Epidemiología y Salud Pública (CIBERESP), 28034 Madrid, Spain; 5 Servicio de Microbiología, Hospital Universitario Ramón y Cajal and Instituto Ramón y Cajal de Investigación Sanitaria (IRYCIS), 28034 Madrid, Spain; juancarlos.galan@salud.madrid.org (J.C.G.); rafael.canton@salud.madrid.org (R.C.); 6 Centro de Investigación Biomédica en Red (CIBER) in Epidemiology and Publich Health, 28029 Madrid, Spain; 7 Red Española de Investigación en Patología en Enfermedades Infecciosas (REIPI), Instituto de Salud Carlos III, 28029 Madrid, Spain; 8 Atenea Research Group, Foundation for the Promotion of Health and Biomedical Research of Valencia Region (FISABIO), 46020 Alicante, Spain; jose.mira@umh.es

**Keywords:** SARS-CoV-2, COVID-19, healthcare workers, risk of transmission, perceived risk

## Abstract

This study aims to identify factors related with SARS-CoV-2 infection in physicians and internal residents during the SARS-CoV-2 pandemic at a tertiary hospital in Spain, through a cross- sectional descriptive perception study with analytical components through two questionnaires directed at professionals working at the Ramon y Cajal University Hospital between February and April 2020. In total, 167 professionals formed the study group, and 156 professionals comprised the comparison group. Seventy percent of the professionals perceived a shortage of personal protective equipment (PPE), while 40% perceived a shortage of hand sanitiser, although more than 70% said they used it properly. Soap was more available and had a higher percentage of correct use (73.6–79.5%) (*p* > 0.05). Hand hygiene was optimal in >70% of professionals according to all five WHO measurements. In the adjusted model (OR; CI95%), belonging to a high-risk specialty (4.45; 1.66–11.91) and the use of public transportation (3.27; 1.87–5.73) remained risk factors. Protective factors were changes of uniform (0.53; 0.32–0.90), sanitation of personal objects before the workday (0.55; 0.31–0.97), and the disinfection of shared material (0.34; 0.19–0.58). We cannot confirm that a shortage or misuse of PPE is a factor in the spread of SARS-CoV-2. Fears and assessments are similar in both groups, but we cannot causally relate them to the spread of infection. The perception of the area of risk is different in both groups, suggesting that more information and education for healthcare workers is needed.

## 1. Introduction

The severe acute respiratory syndrome coronavirus 2 (SARS-CoV-2) was first detected in Wuhan, China, in December 2019, and it has widely spread around the globe [1], becoming a public health emergency of international concern (PHEIC), due to its rapid and hazardous spread to other countries and the need for a coordinated response. On 11 March 2020, the WHO assigned pandemic status to SARS-CoV-2, because of the virus’ characteristics (its high capacity to establish an infection (infectivity), the ability to cause disease (pathogenicity), and the ability to spread (transmissibility)). The basic reproduction number (R0), generally used to determine the transmissibility of a virus, was approximately 2.2 at the start of the pandemic [2]. However, the k-dispersion index is as high as 0.1 in some models, suggesting the important role of individuals and events known as “super-spreaders” [3,4]. Detection through reverse transcription-polymerase chain reaction (RT-PCR) may begin one to two days before the onset of symptoms, with transmission occurring during this period and persisting well after their onset [5,6,7,8,9]. Faecal and airborne transmission by droplet nuclei or aerosols has not been proven outside the experimental setting and still remains controversial [10,11,12,13]. On the other hand, people touch their faces up to 23 times per hour, and 44% of touches are on the mucous membranes [14]. We also know how long human coronaviruses remain active on surfaces [15], and that they are efficiently inactivated in the presence of 62–71% ethanol, 0.1–0.5% sodium hypochlorite, or 2% glutaraldehyde. Consequently, the first measure to implement to stop transmission is proper respiratory hygiene when coughing and sneezing, the second is rigorous hand hygiene and touching our faces as little as possible, the third is observing a personal distance of more than six feet (1.8 metres) [16], and the fourth consists of barrier measures such as the extended standard precautions implemented in hospitals. The general population should also avoid the three Cs—enclosed spaces, crowded places, and close contact—keeping shared objects suitably disinfected [17], including lavatory facilities outside their working environment.

Shortages of materials and disruption in the supply chain have compounded the impact of the SARS-CoV-2 pandemic in all countries. As has happened in other countries, in Spain, the incidence of COVID-19 has varied throughout the country [18]. Among the most severely affected groups in Spain are health workers, with 40,921 cases of COVID-19 reported to Red Nacional de Vigilancia Epidemiológica (RENAVE) by 21 May 2020, accounting for 24.1% of all declared cases [19]. In a study carried out at the Hospital Universitario Doce de Octubre in Madrid, 11.6% of workers tested positive for COVID-19 [20]. COVID-19 has also led to an over-abundance of information, not always accurate, which has spread quickly, causing uncertainty and even states of anxiety (a neologism known as the infodemic). A Google search on the subject returns approximately 4,500,000,000 results (0.64 s), and there have been numerous reports of personal protective equipment (PPE) shortages around the world, with shocking headlines such as The New York Times’ “Health Care Kamikazes: how Spain’s workers are battling Coronavirus, unprotected”, and images of German primary care doctors feeling naked in the presence of SARS-CoV-2 at the URL https://www.blankebedenken.org/ (accessed on 27 April 2020). Although these articles and strategies have a certain sensationalist or provocative lean, Ministerial Departments have indeed been forced into taking exceptional measures regarding the use and prioritisation of PPE in Spain and other countries alike [21,22,23]. In addition, some studies have evidenced healthcare workers having a high perception risk of becoming infected, as well as worries about insufficient PPE and some gaps in knowledge [24,25,26].

**Figure 1:**
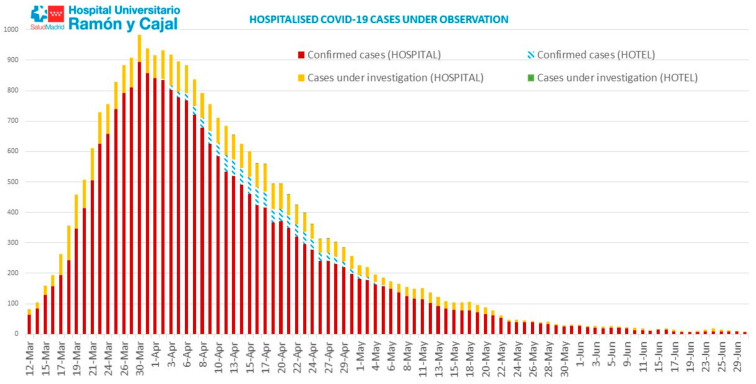
SARS-CoV-2 epidemic curve in HRyC.

On 29 February, the first case was diagnosed at the Hospital Universitario Ramón y Cajal (HR&C). At its normal functioning state, HR&C has 901 active beds. During the first wave of the pandemic, more than 1200 thousand patients were hospitalized simultaneously, more than 1000 being affected by SARS-CoV-2. At the date of writing of this manuscript (August 2020), the hospital has seen 3052 hospitalised patients with suspected or confirmed SARS-CoV2 infection (99 re-admissions), resulting in a total of 2927 discharges, 81% recoveries, 19% deaths; and 0.1% were transferred to other health centres (Figure 1).

The HR&C is a third level hospital, with a high level of specialisation and technology. It holds the seventh position in the ranking of the best hospitals in Spain [27] and is among the best 100 worldwide [28]. It has 5223 workers, of whom 1309 are specialists, including doctors (876) and resident interns (433). Most of these workers are doctors, already specialised or in the process of specialising. On 14 April 2020, there were 446 (8.5%) health workers in HR&C who had tested positive with microbiological detection tests using the RT-PCR technique, 191 of whom (42.83%) were doctors, accounting for 46.28% of the total number of positive RT-PCRs among the centre’s workers. Given the disparity between the weight of this group in the total number of workers and the percentage of affected healthcare workers, it was decided to conduct a survey-based study to analyse the causes of transmission in this group and to implement measures to improve this situation.

The objective of this study was to describe the characteristics, opinions, and activities of hospital physicians and residents during the SARS-CoV-2 pandemic and to identify possible links between COVID-19 infection in this professional group, in order to take measures to halt transmission.

## 2. Materials and Methods

A cross-sectional descriptive perception study with analytical components was conducted through a survey. All the specialists were invited to take part in the study (doctors, pharmacists, biologists, and chemists, including staff in training—the resident intern system of the HR&C (N = 1309). They were allocated to two main sample groups: those who had undergone an RT-PCR assay to detect SARS-CoV-2 (sample 1), and a second group consisting of the remaining 997 workers for whom an RT-PCR [29] had not been requested (sample 2), between 29 February and 14 April 2020.

### 2.1. Survey Instrument, Administration and Sample Selection

Two different and specifically designed questionnaires (A and B) were elaborated for self-administration (available at https://secondvictimscovid19.umh.es/p/download.html (accessed on 27 April 2020)) by the two groups of healthcare workers surveyed.

The variables related to the following areas were collected in questionnaire A, which was sent to group 1: general data (sex, speciality/department, years worked), diagnostic test performed (date of administration, result, motivation for taking the test), self-description of symptoms (date of onset of symptoms, work carried out while symptomatic), usual care administered, care of patients with suspected or confirmed SARS-CoV-2 infection, aerosol generating procedures (AGP), PPE during AGP, type of PPE (mask, gown, eye protection, gloves, makeshift measures), hand hygiene (HH) (hydroalcoholic solution—HAS), soap, WHO moments for HH), preventive measures (HH in the institution before and after the work day, sanitisation of personal objects (mobile phone, keys, usual spectacles, etc.) in the institution before and after the work day, personal distancing, disinfection of shared materials before use, activities in the institution (use of cafeteria, use of common areas, shared office, shared locker room, 24 h shifts and type of bed used); close work and personal contact with a confirmed COVID-19 case; transportation to work; leaving the home; and perception of origin of infection. All the questions referred to the five days before the onset of symptoms or request for RT-PCR if the professional did not present compatible symptoms. In questionnaire B, aimed at sample group 2, variables related to the same areas as in questionnaire A were collected, excluding those related to the diagnostic test. These questions referred to the period from the beginning of the pandemic and the day on which the survey was carried out.

**Figure 2:**
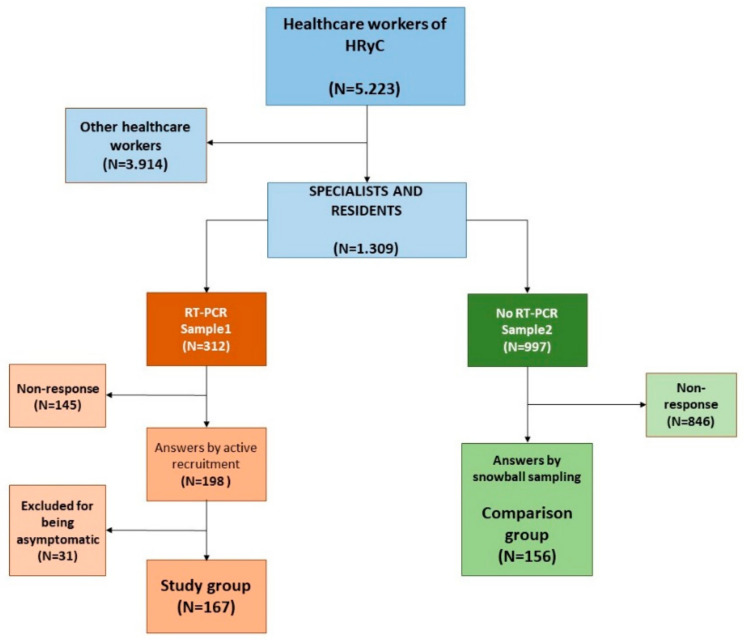
Sample selection.

A total of 312 workers from sample group 1 were contacted by telephone and then received via email the self-administered survey A, with a response rate of 63.5% (198 responses). A total of 167 professionals (84.3% of respondents) presented symptoms compatible with SARS-CoV-2 that motivated a microbiological detection test, forming our study population (study group). All the workers that underwent the test due to other reasons (close contact with a confirmed case, out of fear, or other reasons) were excluded from our study group. A link to questionnaire B was sent to all the departmental heads for distribution among the healthcare professionals, obtaining a response rate of 15.6% (156 responses), thus obtaining our comparison group (Figure 2). The variables were processed and analysed in the same way in both questionnaires.

### 2.2. Data Management and Analysis

The items related to PPE and HH were evaluated on a scale of 0 to 10 (availability and correct use), the optimal level being ≥7. Compliance with preventive measures (HH in the institution before the workday, HH in the institution after the workday, hygiene of personal objects in the institution before and after the workday, social distancing, and disinfection of shared material before use) was scored on a scale of 0 to 10, the optimal level being ≥7. We asked about the activities in the institution in numerical terms, categorising the responses into two groups (yes/no) for analysis purposes. Respondents were asked about their perception of the origin of the infection as free text. The responses were then analysed, grouping them into main categories in which they had appeared in the workers’ responses. For the study, Anaesthesiology and Resuscitation, Pneumology, Intensive Care Medicine, and Emergency Medicine were considered high-risk specialities. The following were considered AGP: endotracheal intubation and extubation, alone or associated with other processes (cardiopulmonary resuscitation, bronchoscopy…), aspiration of respiratory secretions, tracheotomies and fibroscopy, bronchoscopy, BIPAP non-invasive mechanical ventilation, high-flow nasal cannula, treatment with aerosols, sputum induction techniques, autopsies and nasopharyngeal sampling.

Results were exported to and analysed using Stata 16 (Stata Corp. College Station, TX, USA) [30]. The Kolmogorov–Smirnov test was used to test the normality of distribution of continuous variables. Means with standard deviations (SD) were used to describe normally distributed continued variables; non-normal variables were reported as median with interquartile range (IQR). The t-test and Wilcoxon rank-sum test were used to test differences in grouped means and medians, respectively. Categorical variables were compared using the Pearson χ2 or Fisher’s exact test, when appropriate. A value of *p* < 0.05 was considered significant. A univariate analysis was carried out using logistic regression after classifying variables. The significant variables from initial analysis, and those considered relevant according to the current literature and the study purpose, were subjected to multivariate logistic regression. To build the final multivariate regression model, a backward selection method was used. We started by including all the variables meeting the previously mentioned criteria, and then deleting them one by one in order of less significance until no variables could be eliminated without a statistically insignificant loss of fit.

### 2.3. Dissemination of Results

Dissemination to participants and related patient and public communities: the results of this research were reported in a general session at the University Hospital Ramón y Cajal.

### 2.4. Transparency Declaration

The study guarantor affirms that the manuscript is an honest, accurate, and transparent account of the reported study, that no important aspects of the study have been omitted, and that any discrepancies from the study as planned (and, if relevant, registered) have been explained.

### 2.5. Ethics Committee Approval

This study was approved by Ethics Committee of the University Hospital Ramón y Cajal (number approval 240/20).

## 3. Results

The description of our two groups is shown in Table 1. We received 198 responses from the participants in sample 1 (63.5% response rate), 167 (83.5%) of whom showed clinical signs indicative of SARS-CoV-2 infection that justified the diagnostic test, making up our study group. In sample 2, we received 156 responses (a participation rate of 15.6%), forming our comparison group.

Twenty-seven workers (8.4%) were in the high-risk group because of the number of patients treated and the type of procedures performed in their regular practice.

### 3.1. Usual and Risky Clinical Practice 

In the study group, 19 (14.3%) physicians who carry out regular healthcare activities with patients reported having performed some AGP, compared to 8 (6.4%) of the workers in the comparison group (*p* = 0.03).

At the time of the AGP, only five (26.3%) workers in the study group and three (37.5%) in the comparison group wore complete PPE, the waterproof gown being the most frequent PPE lacking during the AGP (seven professionals), followed by the FFP2/3 mask (six professionals) and waterproof goggles/shield (five professionals).

Ninety-two (69.7%) workers in the study group were in direct clinical practice caring for COVID-19 patients, as compared to 112 (89.6%) in the comparison group, regardless of the protective measures used (*p* = 0.019).

Only three workers (1%) reported caring for patients with known COVID-19 infection without protective measures. However, 97 (28%) workers in the study group and 30 (24%) in the comparison group reported treating COVID-19 patients without protective measures when they did not know that these patients were infected (Table 2).

### 3.2. Perceived Risk

The work environment was the most frequently mentioned risk in both groups (78% of the study group versus 89% in the comparison group) followed by the unknown area (8% versus 6%), mixed origin (7% versus 2%), and public transport (0.1% versus 1%). The study population saw the personal realm as risky (6%), while the comparison group did not.

Conversely, the comparison group pointed to the lack of diagnostic tests as a major risk (2%), a category not seen in the study group.

Within the work setting, professionals in the study group perceived contact with infected colleagues to be the greatest risk (27%), followed by the unspecified setting (25%), contact with patients (21%), and lack or poor availability of PPE (15%). However, lack of PPE ranked first in the perception of risk reported by our comparison group (25%), followed by contact with patients (24%), and multifactorial origin (21%), leaving contact with infected colleagues in fourth place (15%). The professionals in the comparison group saw common spaces as a risk more than the colleagues (1% versus 9%) (Table 3). There were no differences between men and women in general perceived risk, nor between areas of perceived risk.

### 3.3. Availability and Use of PPE. Hand Hygiene

The availability and use of PPE are shown in Table 4.

### 3.4. Other Protective Measures

Compliance with other protective measures was higher in the comparison group than in the study group. Although sanitation of personal belongings before the day was below 50% compliance in both groups, in the comparison group, it was 48.7% as opposed to 25.7% in the study group. The biggest difference between both groups was in the disinfection of shared material before use (62% comparison versus 28.7% study). The measure with the highest level of compliance was personal distancing, which was 74.4% in the comparison group, although in the study group it was 46.1% (Table 5).

### 3.5. Activities in the Institution

Concerning the activities carried out in the institution, 80 (47.9%) professionals in the study group and 48.1% of the professionals in the comparison group carried out guards with overnight stays in the centre. A total of 72% of the study group and 59% of the comparison group said they had changed their uniforms; 50% of the study group and 56% of the control group used shared dressing rooms; and 92% of healthcare professionals in the study group and 85% of the comparison group shared an office.

### 3.6. Time Working with Symptoms

One hundred and one (60.5%) healthcare workers in the study group went into work even though they were symptomatic for at least one day. Among the healthcare professionals in our comparison group, 20 (87%) continued to work for one day or more while experiencing symptoms (Table 6).

Sickness presenteeism was more frequent in women in both our groups. Globally, 72% of women presenting symptoms went into work, compared to 53% of men (RR 1.3; *p* < 0.001).

### 3.7. Close Contact with Suspected/Confirmed Cases 

A total of 107 (64%) of the professionals in the study group reported close contact with a health worker confirmed to be infected with SARS-CoV-2, compared to 116 (74%) of the professionals in the comparison group (*p* = 0.046).

Thirty-nine healthcare workers in the study group (23%) and 40 (25%) of the comparison group reported close personal contact with a confirmed case of COVID-19 infection (*p* = 0.633).

### 3.8. Public Transport

A total of 102 (61%) professionals in the study population reported going to work mostly by private transport or on foot, compared to 127 (81.4%) professionals in the comparison population (*p* < 0.05).

### 3.9. Protective and Risk Factors

Individually, the variables that were statistically associated with belonging to the study population are shown in Table 7. Sanitation of personal objects before and after the day, disinfection of shared material, frequent changes of uniform, and maintaining personal distance measures showed a protective effect. Use of public transport, carrying out AGP, and belonging to one of the specialities considered to be at high risk were identified as risk factors.

When building the adjusted model, belonging to a high-risk speciality and the use of public transportation were still risk factors.

Protective factors included changes of uniform, sanitation of personal objects before the workday, and the disinfection of shared material (Table 7).

## 4. Discussion

The response rate differed greatly between groups, basically due to the different recruitment strategies used. Workers in the group that underwent microbiological tests were recruited actively. Each worker was contacted by phone and sent a link to questionnaire A after obtaining their consent to take part. In the group of workers without an RT-PCR test (comparison group), a snowball sampling method was used through the Heads of Service of the HR&C. Therefore, the response rate is lower (63.5% versus 15.6%).

Regarding the diagnostic tool, the RT-PCR technique is the gold standard for in vitro diagnosis of SARS-CoV-2 infection. However, none of the techniques give a 100% precise result. A published systematic review described a false negative rate of up to 29% (3–29%) [31]. Therefore, it is essential to consider clinical suspicion, the existence of alternative diagnoses, and the epidemiological and radiological context of the patients in the diagnostic equation, particularly in patients where the RT-PCR is negative [32]. At the time of our study, the Community of Madrid had an epidemiological context of high community transmission of SARS-CoV-2, with a high weight of health professionals among those infected [33]. We evaluated the clinical signs compatible with COVID-19 infection in our professionals (cough, fever, dyspnoea), and took into account the occupational risk of working in a healthcare centre, in a context where about 20% of reported cases in Spain occurred in health personnel. Between 24 and 28 of April, shortly after the completion of this study, the Microbiology Service of the Ramón y Cajal Hospital carried out a seroprevalence study among all the centre’s personnel (healthcare and non-healthcare workers), obtaining a conversion prevalence of 24% (unpublished data), at a time when the seroconversion prevalence in the Community of Madrid was 11.3% and 5% in Spain, thus supporting the concept of occupational risk for healthcare workers. We could not compare the results of our survey with those of the Microbiology Department due to the anonymity of the responses in our study. Nevertheless, taking into account the weight of healthcare workers among the infected and the seroconversion prevalence rate at HR&C, we consider that our case definition was accurate, as it included the professionals whose request for RT-PCR for SARS-CoV-2 was motivated by the presence of compatible symptoms, regardless of the outcome.

A great majority of our professionals think the work setting is the area of origin of their infection, or the area with greater risk of becoming infected. Among these professionals, up to 22% of our comparison group and 11% of our study group consider the shortage of PPE as the origin of the infection. In addition, none of the professionals in our comparison group perceived the personal environment as the main potential source of infection. However, we only explored the area of perceived risk, and the study does not look into a broader sense of risk perception and how it might influence the actions and activities of our population. People who have had contact with the disease (study group) usually perceive more risk, or have a more accurate risk perception in comparison to those who have not been infected [34]. Additionally, the beliefs about one’s probability of becoming infected has been shown to predict greater self-reported engagement in protective behaviour in some studies [35]. A more general approach regarding this subject might be useful to better understand and address professionals’ fears and experiences.

The results obtained in our study show that a shortage or misuse of PPE cannot be blamed for accelerating the spread of SARS-CoV-2, although 70% of participants declared having experienced shortages. The time distribution of the RT-PCR in our healthcare professionals was homogeneous during the study period, unlike the curve of patients admitted to our hospital, which is concentrated between 25 March and 7 April. This leads us to believe that although it is possible that a lack of PPE was a factor in the transmission of the infection among our staff, there were other factors at play, related mainly to the disinfection and distancing measures adopted. The workers taking part clearly perceived a low availability of PPE, although it is difficult to assess real scarcity in relation to the centre’s needs during the pandemic. Furthermore, in a high-pressure atmosphere such as that experienced in Spain, and the high accompanying infodemics, the beliefs of healthcare professionals about what elements should make up PPE may not be compatible with the real recommendations issued by official bodies based on risk. However, this aspect has not been addressed in this study. Knowledge, perception, and experiences are crucial in the establishment of preventive behaviours and measures. It would be interesting to explore the prior knowledge and beliefs of healthcare professionals in a future study in order to develop an effective communication strategy that would improve adherence to precautions and worker safety, especially with the threat of a second wave in the near future [36,37]. We did not do so in our study, since the main objective of our inquiry was to establish any correlations with basic and rapid action measures, in order to halt transmission among our professionals.

Along with the proper use of PPE, hygiene-based measures, as well as standard precautions and precautions based on the transmission of microorganisms, are essential for infection control within the hospital setting [38]. In this regard, the protective effect of disinfection measures, both for shared material and personal objects, is of particular importance in our study, coinciding with the data available in other publications during the pandemic [39]. Likewise, the protective effect of stripping down and donning a clean uniform is striking.

Sickness presenteeism is defined as working while being sick. Twenty workers from the comparison group and 101 from the study group worked at least one day with symptoms compatible with SARS-CoV-2. This information is striking since, given the appearance of symptoms suggestive of SARS-CoV-2, professionals were urged to stay at home until the case was ruled out or confirmed. However, this could be explained by the fact that, as described in the literature, medical workers generally take less sick leave than other health professional groups. In addition, work presenteeism was more prevalent in women. This finding is consistent with the current literature, where women are found to more often go to work when sick than men, in relation with gender stereotypes such as women being taught to focus on others more than on themselves, thus justifying their presence in the workplace even when feeling ill [40]. These facts are, in our opinion, another area of improvement in the medical profession.

According to our results, belonging to one of the specialities that we have considered high risk (Pneumology, Intensive Care Medicine, Anaesthesia and Resuscitation, Emergency Medicine) is related to belonging to our study group. Despite the controversy generated in scientific and social circles regarding the transmission mechanisms of SARS-CoV-2, in addition to transmission by droplets, airborne transmission of SARS-CoV-2 is plausible when certain procedures that generate aerosols are performed on infected patients. This is in line with our results, since having performed AGP is presented as a risk factor for belonging to the study population. However, the analysis of these data requires caution due to the low number of professionals in our sample who reported having performed an AGP.

It should be noted that our data are based on professionally referred responses, with the risk of bias inherent in survey-based studies. In addition, the administered questionnaires are not validated tools, but designed specifically for the purpose of the study. However, despite these limitations, the readability and legibility of the questionnaires were assured. This research was conducted during the first wave and focused on the comparisons of perceived risk factors related to work setting, personal environment, and use of public transport. Future research should analyse the influence of individual differences and psychological factors in the perception of risk. Despite this, our results are supported by the available literature and evidence that point to standard precautions and basic hygiene measures (change of uniform, disinfection of surfaces and shared materials), as well as personal distancing, as key measures to prevent transmission among professionals in the hospital setting.

**Figure 3:**
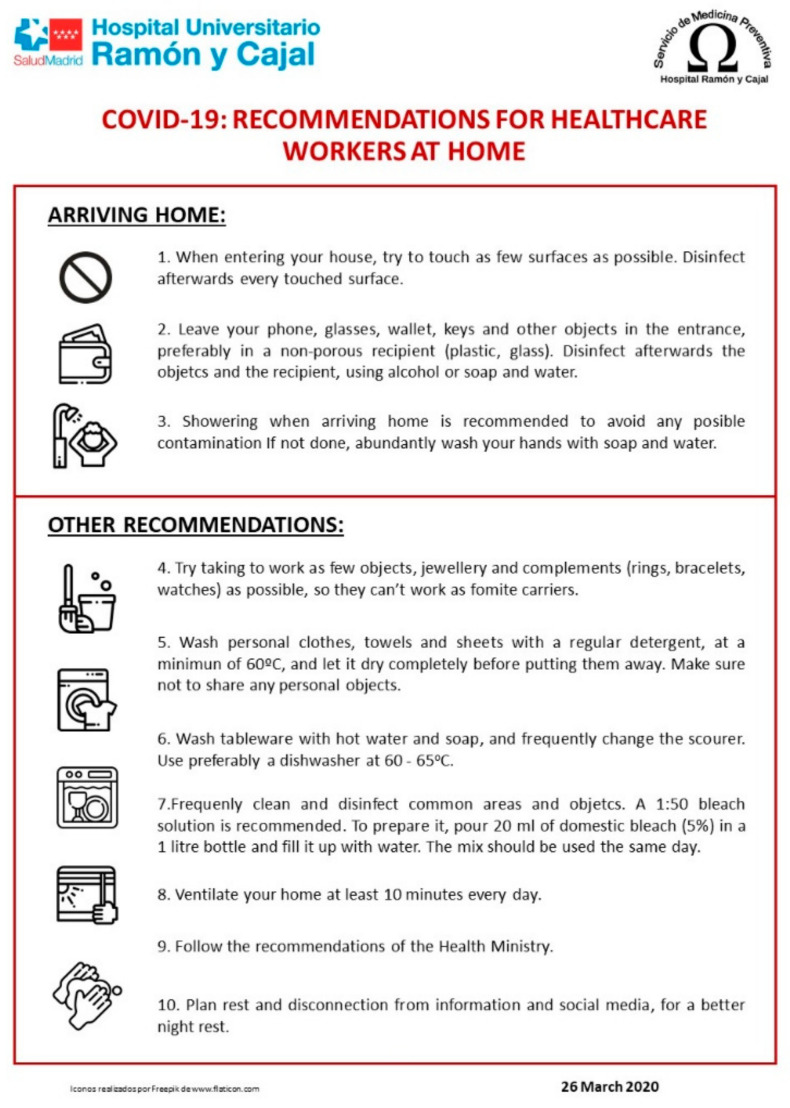
Recommendations for health professionals.

Therefore, among the measures implemented by the Preventive Medicine and Public Health Service of the Hospital Universitario Ramón y Cajal, the following stand out: more frequent disinfection of common areas (on-call rooms, offices, shared stays, etc.), training in waste management and circuits of hospitalisation areas, and hygiene recommendations for professionals before and after the workday (Figure 3). Additionally, to alleviate the shortage of hand sanitiser, the Pharmacy Service started producing hand sanitiser for the centre, producing 2068 litres during the pandemic. Concerning the PPE, a feasibility study was conducted for reprocessing FFP2 masks, although in the end, it was not necessary to implement it. However, it was necessary to reprocess liquid-resistant gowns at the Sterilisation Centre (20,202 reprocessing in total through the period). This last measure helped to mitigate the shortage of some elements of PPE.

It is not clear whether these results are transferable to healthcare settings in other regions. As far as our knowledge goes, there are no other studies of the same nature performed in Spain, thus comparability cannot be established. On the other hand, the extreme pressure caused by COVID-19’s healthcare demands was transversal to all of the Madrid region. We may infer, by this logic, that these results can be extrapolated to other healthcare facilities in the area, though more studies should be performed.

We have not addressed in this study the influence of potential vaccination against COVID-19. This aspect was not included in our questionnaires, since the study was developed at an early stage in the pandemic, when there was no evidence of a vaccine being available in the foreseeable future. It would be interesting to evaluate if vaccination (willingness or hesitancy towards getting vaccinated) has any influence on the activities and perceived risk of healthcare workers towards COVID-19.

Feelings of perceived life threat and anxiety that may translate into psychological impact and distress have been associated with public health disasters such as pandemics [41,42]. This is a future line of study to consider, in order to further shed light on the implications of our findings.

## 5. Conclusions

During this outbreak, the concern about becoming infected has become widespread, particularly during the first wave when this study was conducted. Adequate and timely information and training are essential to ensure the responsiveness of the healthcare workforce. The perception of risk through word-of-mouth does not always correspond to the danger (actual risk). A majority of healthcare professionals experienced shortages during the first wave, which probably increased their perception of risk. Fears and assessments were similar in both comparison groups, but we cannot causally relate them to the spread of the infection among professionals.

Although our results in hand hygiene compliance are high, the reinforcement of basic control infection key messages is always necessary. Even when there is no solid evidence of aerosol transmission, performing AGP or belonging to a risky speciality increases the perception of the risk of infection.

The professionals in the study group (RT-PCR motivated by SARS-CoV-2-compatible symptoms) consider that contact with colleagues with COVID-19 infection or with patients is responsible for their disease 48% of the time, thus perceiving a higher risk of transmission in the workplace. This point should be addressed by policy makers and hospital management teams, in order to give more information and education on preventive measures in the workplace. In addition, measures to disinfect shared material, change one’s uniform, and maintain the hygiene of personal objects show a protective effect and should be a major concern for everyone implicated in the healthcare setting during the SARS-CoV-2 pandemic.

## Figures and Tables

**Table 1:** Group description.

	Study Group	Comparison Group	*p*-Value
Median years of experience (interquartile range)	12 years (4, 22)	14 years (5, 26)	
Sex			0.3
Men N(%)	79 (47.3)	65 (41.7)	
Women N(%)	88 (52.7)	91 (58.3)	
Working group			0.07
Resident Intern N(%)	52 (31.1)	35 (22.4%)	
Specialist physician N(%)	115 (68.9)	121 (77.6%)	
High-risk specialists ^1^ N(%)	20 (12)	7 (4.5)	0.01

^1^ Anaesthesiology and Resuscitation, Pneumology, Intensive Care Medicine, and Emergency Medicine.

**Table 2:** Care of suspected/confirmed COVID-19 patients and the use of protective measures (%).

	Study Group N(%)	Comparison Group N(%)	OR (IC95; *p*-Value)
Yes, I already knew and used protective measures	40 (30.3)	64 (51.2)	0.41 (0.24–0.71; 0.0006)
Yes, I already knew but I did not use protective measures	1 (0.8)	2 (1.6)	0.47 (0.00–9.14; 0.6135) ^1^
Yes, but I did not know, and I did not use protective measures	37 (28)	30 (24)	1.23 (0.68–2.25; 0.4620)
Yes, but I did not know, and, despite everything, I used protective measures	14 (10.6)	16 (12.8)	0.81 (0.35–1.86; 0.5841)
I have not cared for any confirmed or suspected patients	40 (30.3)	13 (10.4)	3.75 (1.82–8.07; 0.0001)
Total	132 (100)	125 (100)	

^1^*p*-value according to Fisher’s exact test.

**Table 3:** Healthcare professionals’ perception of risk.

	Study Group N (%)	Comparison Group N (%)	*p*-Value	Work Setting			
					Study Group N (%)	Comparison Group N (%)	*p*-Value
Work setting	130 (77.8)	139 (89.1%)	0.1089	Colleagues	35 (26.9)	19 (13.7)	0.0067
Unknown setting	14 (8.4)	9 (5.8%)	0.4331	Patients	27 (20.8)	34 (24.5)	0.2599
Personal environment	11 (6.6)			Mixed	15 (11.5)	29 (20.9)	0.0388
Mixed area	11 (6.6)	3 (1.9%)	0.0541 ^1^	PPE	19 (14.6)	35 (25.2)	0.1202
Public transport	1 (0.6)	1 (0.6%)		Common spaces	1 (0.8)	13 (9.3)	0.0015 ^1^
Lack of testing		4 (2.6%)		Not specified	33 (25.4)	9 (6.4)	<0.0001

^1^*p*-value according to Fisher’s exact test.

**Table 4:** Degree of availability and proper use of personal protective equipment (PPE) and hand hygiene products and performance.

		Study Group N (%)		Comparison Group N (%)		
		Optimal (≥7)	Suboptimal (<7)	Optimal (≥7)	Suboptimal (<7)	*p*-Value
Mask	Availability	38 (28.8%)	94 (71.2%)	35 (28%)	90 (72%)	0.8887
	Correct use	74 (56.1%)	58 (43.9%)	70 (56%)	55 (44%)	0.9922
Goggles	Availability	37 (28%)	95 (72%)	42 (33.6%)	83 (66.4%)	0.3334
	Correct use	46 (34.9%)	86 (65.1%)	58 (46.4%)	67 (53.6%)	0.0593
Gloves	Availability	98 (74.2%)	34 (25.8%)	97 (77.6%)	28 (22.4%)	0.5292
	Correct use	103 (78%)	28 (22%)	103 (82.4%)	22 (17.6%)	0.3792
Gowns	Availability	28 (21.2%)	104 (78.8%)	22 (17.6%)	103 (82.4%)	0.4641
	Correct use	51 (38.7%)	81 (61.3%)	59 (47.2%)	66 (52.8%)	0.9052
Hand sanitiser	Availability	77 (58.3%)	55 (41.7%)	72 (57.6%)	53 (42.4%)	0.9052
	Correct use	102 (77.3%)	30 (22.7%)	95 (76%)	30 (24%)	0.8095
Soap	Availability	100 (75.8%)	32 (24.2%)	94 (75.2%)	31 (24.8%)	0.9173
	Correct use	105 (79.6%)	27 (20.4%)	92 (73.6%)	33 (26.4%)	0.2600
Before touching the patient	Compliance	98 (74.2%)	34 (25.8%)	92 (73.6%)	33 (26.4%)	0.9067
Before the aseptic technique	Compliance	106 (80.3%)	26 (19.7%)	101 (80.8%)	24 (19.2%)	0.9199
After exposure to fluids	Compliance	111 (84.1%)	21 (15.9%)	107 (85.6%)	18 (14.4%)	0.7360
After contact with the patient	Compliance	110 (83.3%)	22 (16.7%)	103 (82.4%)	22 (17.6%)	0.8427
After the environment	Compliance	94 (71.2%)	38 (28.8%)	99 (79.2%)	26 (20.8%)	0.1378

**Table 5:** Compliance with other protective measures.

	Study Group N (%)		Comparison Group N (%)		
	Optimal (≥7)	Suboptimal (<7)	Optimal (≥7)	Suboptimal (<7)	*p*-Value
Hand hygiene before the workday	127 (76.1%)	40 (23.9%)	130 (83.3%)	26 (16.7%)	0.1034
Hand hygiene after the workday	137 (82.1%)	30 (17.9%)	132 (84.6%)	24 (15.4%)	0.5342
Personal object sanitation before the start of the day	43 (25.7%)	124 (74.3%)	76 (48.7%)	80 (51.3%)	<0.0001
Personal object sanitation at the end of the work day	69 (41.3%)	98 (58.7%)	99 (63.5%)	57 (36.5%)	0.0001
Personal distancing	77 (46.1%)	90 (53.9%)	113 (74.4%)	43 (27.6%)	<0.0001
Prior disinfection of shared material	48 (28.7%)	119 (71.3%)	97 (62.2%)	59 (37.8%)	<0.0001

**Table 6:** Frequency of days working with symptoms.

	Study Group N (%)	Comparison Group N (%)	*p*-Value
0 days	66 (39.5%)	3 (13.1%)	
1–3 days	68 (40.8%)	10 (43.4%)	
4 or more days	33 (19.7%)	10 (43.4%)	
Total	167 (100%)	23 (100%)	0.011

**Table 7:** Association of variables as risk/protective factors.

	OR (DE)	*p*-Value	CI95%
**Univariate Regression**			
Public transport	2.79 (0.72)	<0.0001	1.68–4.64
Having carried out AGP	2.45 (1.08)	0.042	1.03–5.84
Risk speciality	2.9 (1.31)	0.019	1.18–7.05
Personal object sanitation before the workday	0.37 (0.09)	<0.0001	0.23–0.58
Personal object sanitation at the end of the workday	0.41 (0.09)	<0.0001	0.26–0.63
Personal distancing	0.33 (0.7)	<0.0001	0.20–0.51
Disinfection of shared material	0.24 (0.05)	<0.0001	0.15–0.39
Change of uniform	0.55 (0.13)	0.013	0.35–0.88
Close contact with a sick SARS-CoV-2 confirmed health worker	0.61 (0.15)	0.045	0.38–0.99
**Multivariate regression**			
Risk speciality	4.45 (2.23)	0.003	1.66–11.91
Public transport	3.27 (0.94)	<0.0001	1.87–5.73
Personal object sanitation before the workday	0.55 (0.16)	0.040	0.31–0.97
Disinfection of shared material	0.34 (0.09)	<0.0001	0.19–0.58
Change of uniform	0.53 (0.14)	0.019	0.32–0.90

## Data Availability

The data presented in this study are available on request from the corresponding author.

## Supplementary Materials

The following are available online at https://www.mdpi.com/1660-4601/18/6/2838/s1, Questionnaires A,B: Questionnaire for Healthcare Professionals during the SARS-COV-2 Pandemic.
