# Supplementary material for: Activities and Perceived Risk of Transmission and Spread of SARS-CoV-2 among Specialists and Residents in a Third Level University Hospital in Spain
Source: Int J Environ Res Public Health. 2021 Mar 10;18(6):2838. doi: 10.3390/ijerph18062838 (PMC7998370; doi:10.3390/ijerph18062838)
Supplement: Supplementary file 1 [file ijerph-18-02838-s001.zip › Questionnaire A.pdf]

QUESTIONNAIRE FOR HEALTHCARE PROFESSIONALS DURING THE SARS-CoV-2 PANDEMIC

Sex

- Male
- Female

Service/Specialties

- Allergology
- Anesthesiology
- Anatomical pathology
- Clinical Biochemistry
- Pediatric Cardiology
- Cardiology
- Cardiac Surgery
- General and Digestive Surgery
- Oral and maxillofacial surgery
- Plastic surgery
- Thoracic surgery
- Vascular surgery
- Palliative Care
- Dermatology
- Endocrinology and nutrition
- Infectious Diseases
- Pharmacology
- Gastroenterology
- Genetic
- Geriatrics
- Gynaecology
- Hematology
- Immunology
- General Medicine
- Intensive Care Medicine
- Internal Medicine
- Preventive Medicine and Public Health
- Microbiology
- Nephrology
- Neumology
- Neurosurgery
- Clinical Neurophysiology
- Ophthalmology
- Medical oncology
- Radiotherapy
- Otorhinolaryngology
- Occupational Health
- Clinical psychology
- Pediatrics

- Psychiatry
- Radiophysics
- Radiology
- Rehabilitation
- Rheumatology
- Traumatology
- Emergency Medicine
- Urology

EXPERIENCE

- Years working as an attending physician
- Years working as a resident

TEST

Result of the laboratory SARS-CoV-2 diagnostic test? Positive/Negative

Day of testing: dd/mm/yyyy

Reason for SARS-CoV-2 test:

- Probable case. Patient with compatible clinical symptoms with SARS-CoV-2 infection (fever, cough, difficult breathing)?
- Possible case. Patient with compatible clinical symptoms with upper/lower respiratory tract infection?
- Asymptomatic case with close contact with a positive case.
- Out of fear of being positive and infecting my close contacts.
- Others

SYMPTOMS

Since the start of the SARS-CoV-2 pandemic, have you experienced any symptoms compatible with Covid-19 infection? Yes/No

Date of symptoms onset: dd/mm/yyyy

How many days did you work with symptoms?

ASSISTENTIAL ACTIVITY

In the 5 days prior to your onset symptom date (or the test if you are an asymptomatic contact), have you participated in any clinical patient-related activities (ER, consultation, hospitalization…)? Yes/No

Since the start of the SARS-CoV-19 pandemic, have you attended to any infected (confirmed or suspected) patients with SARS-CoV-2?

a. Yes, I was aware of the patient’s condition, so I made use of all the protection measures established by my institution.

b. Yes, I was aware of the patient's condition, but I didn't use the protection measures established by my institution
c. Yes, but I was not aware of the patient's condition, so I didn't use the protection measures established by my institution.
d. Yes, even though I was not aware of the patient's condition, I used the protection measures established by my institution
e. No, I haven't attended on any patient with suspected/confirmed Covid-19 infection.

During this period, have you performed any aerosol generating procedures?

When performing an aerosol generating procedure, where you wearing all the elements of the personal protective equipment (PPE)?

a. Yes, I was wearing a complete PPE
b. No, I wasn't wearing a complete PPE

Which elements of the PPE were you wearing when performing an aerosol generating procedure?

PROTECTIVE MEASURES

In a scale from 0 to 10 (0 being the worst option, and 10 being the best option), how would you describe the use of these protective measures?

Surgical or FPP2 mask

- Availability: 0-10
- Use when necessary following the institution's recommendations: 0-10
- Perception of a correct use: 0-10

Safety goggles/face shields

- Availability: 0-10
- Use when necessary following the institution's recommendations: 0-10
- Perception of a correct use: 0-10

Nitrile gloves

- Availability: 0-10
- Use when necessary following the institution's recommendations: 0-10
- Perception of a correct use: 0-10

Disposable and fluid resistant gowns:

- Availability: 0-10
- Use when necessary following the institution's recommendations: 0-10
- Perception of a correct use: 0-10

Other non-homologated measures:

HAND HYGIENE PRODUCTS

In a scale from 0 to 10 (0 being the worst option, and 10 being the best option), how would you describe the use of these hand hygiene products and activities?

Hydroalcoholic Solution

- Availability: 0-10
- Use when necessary following the institution's recommendations: 0-10
- Perception of a correct use: 0-10

Hand soap:

- Availability: 0-10
- Use when necessary following the institution's recommendations: 0-10
- Perception of a correct use: 0-10

5 Moments for Hand Hygiene

Before touching a patient: 0-10

Before clean/aseptic procedures: 0-10

After body fluid exposure/risk: 0-10

After touching a patient: 0-10

After touching patient surroundings: 0-10

PREVENTION MEASURES

In a scale from 0 to 10 and since the start of the SARS-CoV-2 pandemic, which of these measures have you performed and how?

- Hand hygiene in your institution before your workday: 0-10
- Hand hygiene in your institution after your workday: 0-10
- Hygiene of personal objects (keys, mobile phone…) in your institution before your workday: 0-10
- Hygiene of personal objects (keys, mobile phone…) in your institution after your workday: 0-10
- Social distancing (at least 1 meter) in the workplace: 0-10
- Disinfection of shared materials (computers, pager…) before use: 0-10

ACTIVITIES IN THE INSTITUTION

Since the since the start of the SARS-CoV-2 pandemic:

- How many times did you use the hospital's cafeteria?
- How many times did you have a meal in a common area (office, common room…)?
- How many times did you go to the laundry facilities to change the work uniform?
- How many times did you share a changing room?
- How many DAYS did you share a work office?

- How many 24-hour on-call shifts did you have?
  - 0
  - 1-5
  - 6-10
  - >10
- If you had any 24-hour on-call shifts, what type of bed did you use?
  - Shared bed
  - Single bed

OTHER ACTIVITIES AND CONTACT

Since the since the start of the SARS-CoV-2 pandemic, have you had any known close contact* with a confirmed or suspected SARS-CoV-2 patient out of the workplace environment (household…)? Yes/No

*Close contact: providing care without recommended PPE or a possible breach of PPE; household relatives; having had face-to-face contact with a COVID-19 case within 2 meters for more than 15 minutes.

Since the since the start of the SARS-CoV-2 pandemic, have you had any known close contact* with a confirmed or suspected SARS-CoV-2 patient in the workplace environment (referring to coworkers and not patients)? Yes/No

*Close contact: providing care without recommended PPE or a possible breach of PPE; household relatives; having had face-to-face contact with a COVID-19 case within 2 meters for more than 15 minutes.

Since the since the start of the SARS-CoV-2 pandemic, what is your main mode of transport to work?

- Walking
- Bicycle
- Motorcycle
- Private car
- Shared car
- Public transport (subway, bus, train…)
- Taxi

Have you gone to any activities other than work (grocery shopping…)? Yes/No

In your opinion, how did you become infected with SARS-CoV-2?

In your opinion, which situations put you at the most risk of SARS-CoV-2 infection?
